# PhOTO Zebrafish: A Transgenic Resource for *In Vivo* Lineage Tracing during Development and Regeneration

**DOI:** 10.1371/journal.pone.0032888

**Published:** 2012-03-14

**Authors:** William P. Dempsey, Scott E. Fraser, Periklis Pantazis

**Affiliations:** 1 Division of Biology, Beckman Institute (139-74), California Institute of Technology, Pasadena, California, United States of America; 2 ETH Zurich, Department of Biosystems Science and Engineering, Basel, Switzerland; National Cancer Institute, United States of America

## Abstract

**Background:**

Elucidating the complex cell dynamics (divisions, movement, morphological changes, etc.) underlying embryonic development and adult tissue regeneration requires an efficient means to track cells with high fidelity in space and time. To satisfy this criterion, we developed a transgenic zebrafish line, called PhOTO, that allows photoconvertible optical tracking of nuclear and membrane dynamics *in vivo*.

**Methodology:**

PhOTO zebrafish ubiquitously express targeted blue fluorescent protein (FP) Cerulean and photoconvertible FP Dendra2 fusions, allowing for instantaneous, precise targeting and tracking of any number of cells using Dendra2 photoconversion while simultaneously monitoring global cell behavior and morphology. Expression persists through adulthood, making the PhOTO zebrafish an excellent tool for studying tissue regeneration: after tail fin amputation and photoconversion of a ∼100µm stripe along the cut area, marked differences seen in how cells contribute to the new tissue give detailed insight into the dynamic process of regeneration. Photoconverted cells that contributed to the regenerate were separated into three distinct populations corresponding to the extent of cell division 7 days after amputation, and a subset of cells that divided the least were organized into an evenly spaced, linear orientation along the length of the newly regenerating fin.

**Conclusions/Significance:**

PhOTO zebrafish have wide applicability for lineage tracing at the systems-level in the early embryo as well as in the adult, making them ideal candidate tools for future research in development, traumatic injury and regeneration, cancer progression, and stem cell behavior.

## Introduction

Extensive migratory events, morphological changes, and cell divisions coordinated by cell signaling drive complex vertebrate patterning events during embryonic development [1] as well as during epimorphic tissue regeneration [2]. Lineage tracing – the process of tracking a cell and its progeny in space and time as it moves from an early to a specified state – provides an overall picture of the coordinated events required to set up or repair a proper body plan. Two major considerations are required for this tracking strategy: first, a precise, indelible mark must be placed within cells of interest at an early stage of development or regeneration; and second, after sufficient time, the final location of labeled cells and their progeny must be scored accurately [3]. Thus, techniques capable of monitoring long-term behavior (i.e. movements as well as morphology) in precise cell locations are key to tease out the timing and cellular events controlling general cell fate decisions *in vivo*.

Most techniques for lineage tracing can be split into two general categories, (1) sparse/partial and (2) global cell labeling and tracking. Sparse cell labeling methodologies include traditional lineage tracing analyses involving early stage dye injection (e.g. fluorescein, etc.), followed by fixed sample analysis or live cell tracking after sufficient developmental time [3]. More recent sparse labeling modalities take advantage of genetically encoded means for generating random transgenic mosaics expressing cell-targeted fluorescent proteins (FPs) – for example, using random gene insertion events [4], [5], drug induced recombination [6], [7] or heat shock promoters [8]. Other partial cell labeling techniques revolve around tissue specific promoter driven FP expression to isolate only cells in a particular tissue of interest [9], [10], [11], [12], [13]. The second variety, global cell labeling, is most often achieved by ubiquitous nuclear FP expression driven by constitutive promoter sequences [14], [15].

These two techniques have opposing benefits and disadvantages that limit their effectiveness as lineage tracing tools. Sparse/partial methods simplify the tracking problem by focusing solely on cells of interest, though the data lacks context with other cell types in a given tissue environment. In contrast, global methods supply cell behavior data in context to every cell in the organism at the expense of requiring sophisticated volumetric imaging and algorithms for cell segmentation and tracking. An attractive lineage tracing tool should combine the strengths of both and should be directly applicable for studies of early development as well as adult tissue regeneration.

Here, we report a set of transgenic zebrafish lines that we named PhOTO (photoconvertible optical tracking of…), which meets the challenges of long-term lineage tracing in both early development as well as adult epimorphic tissue regeneration. These lines combine the benefits of global and sparse imaging approaches for lineage analysis. Whole organism imaging and tracking is enhanced by the multicompartment (nuclear and membrane) expression of spectrally distinct FPs from a single coding sequence, providing better accuracy for segmentation and tracking of cells *in vivo*. Additionally, with the aid of optically induced photoconversion, long-lasting sparse cell labeling is easily achieved, allowing high signal-to-noise tracking of a subset of cells in space and time at any point during the lifetime of the zebrafish.

## Results

### Establishing the PhOTO-N and PhOTO-M transgenic lines

**Figure 1:**
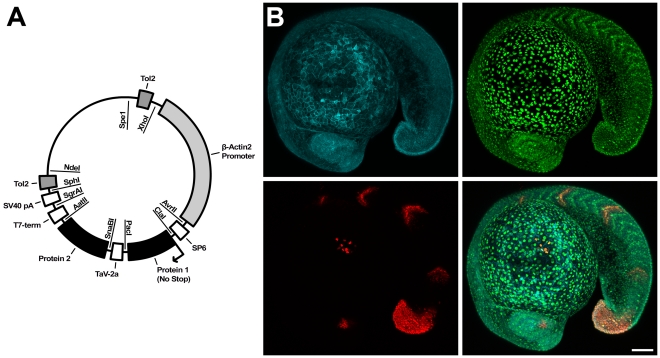
Description of PhOTO Vector and PhOTO Transgenics. (**A**) Depicted is a schematic of the general PhOTO vector. The major components of the vector, including the promoter, the TaV *2A* sequence, the *Tol2* transposable elements, and the protein locations are indicated on the outside of the plasmid circle. Each vector was designed such that each of the components (e.g. the promoter, the FPs, etc.) may be easily switched out for alternate protein fusions, etc. using the listed restriction enzymes (inside the circle) and an appropriate subcloning procedure. Note that the sizes of the blocked regions indicating coding sequences are not to scale. (**B**) Representative heterozygous PhOTO-N expression in an 18–19 hour post fertilization F1 transgenic zebrafish embryo. The top left panel of (B) depicts memb-Cerulean (blue); the top right panel depicts unconverted H2B-Dendra2 (green); the bottom left panel depicts photoconverted H2B-Dendra2 (red) in 4 somites, the tip of the tail, a subset of cells in the eye, and a subset of cells atop the yolk; and the bottom right panel depicts a merged image of all three colors. Scale bar is 100µm.

In order to best address the requirements for long-term imaging of complex vertebrate development and regeneration, we constructed the PhOTO vector (**Figure 1A**), which allows for constitutive *β*-*actin2* [16] driven expression of two FPs – green-to-red photoconvertible Dendra2 [17] and the blue FP Cerulean [18] – targeted to either (1) the nucleus of the cell by means of an H2B fusion or (2) the membrane (“memb”) via a palmitoylation and myristoylation fatty acid substrate sequence included at the N-terminus of the protein [19]. Self-cleavage of a highly efficient *Thosea asigna* virus (TaV) *2A* sequence between the two FPs at the ribosome separates the two proteins to their respective target locations within the cell at a 1∶1 stoichiometric ratio [20]. Two stable transgenic lines (*Tg(βactin2:memb*-*Cerulean-2A-H2B-Dendra2)^pw1^* and *Tg(βactin2:memb-Dendra2-2A-H2B-Cerulean)^pw2^*) were established after Tol2 mediated [21] genome integration. PhOTO-N zebrafish constitutively express H2B-Dendra2 to label all nuclei and memb-Cerulean to label all membranes, and the complementary PhOTO-M zebrafish line expresses H2B-Cerulean and memb-Dendra2. Efficient cleavage and proper FP localization were verified by western blot (**Figure S1**) and confocal microscopy (**Figure 1B**). Founders were established for each line (7 for PhOTO-N; 5 for PhOTO-M), and only offspring with ubiquitous, bright FP expression in all cells were considered for this analysis.

### PhOTO-N enables precise nuclear tracking during early development

Since nuclear tracking has become standard practice for developmental lineage tracing [14], [15], we illustrated the simplicity of targeting down to the level of a single cell during the dynamic period of gastrulation in the PhOTO-N line (**Figure S2** and **Videos S1, S2**). Our approach only requires semi-automated image analysis (see methods), and we segmented and tracked the photoconverted H2B-Dendra2 nuclei in time through ∼6 hours of development. Simultaneous collection of memb-Cerulean and unconverted nuclear H2B-Dendra2 fluorescence data from all surrounding cells gives context to the behavior of the photoconverted nuclei.

**Figure 2:**
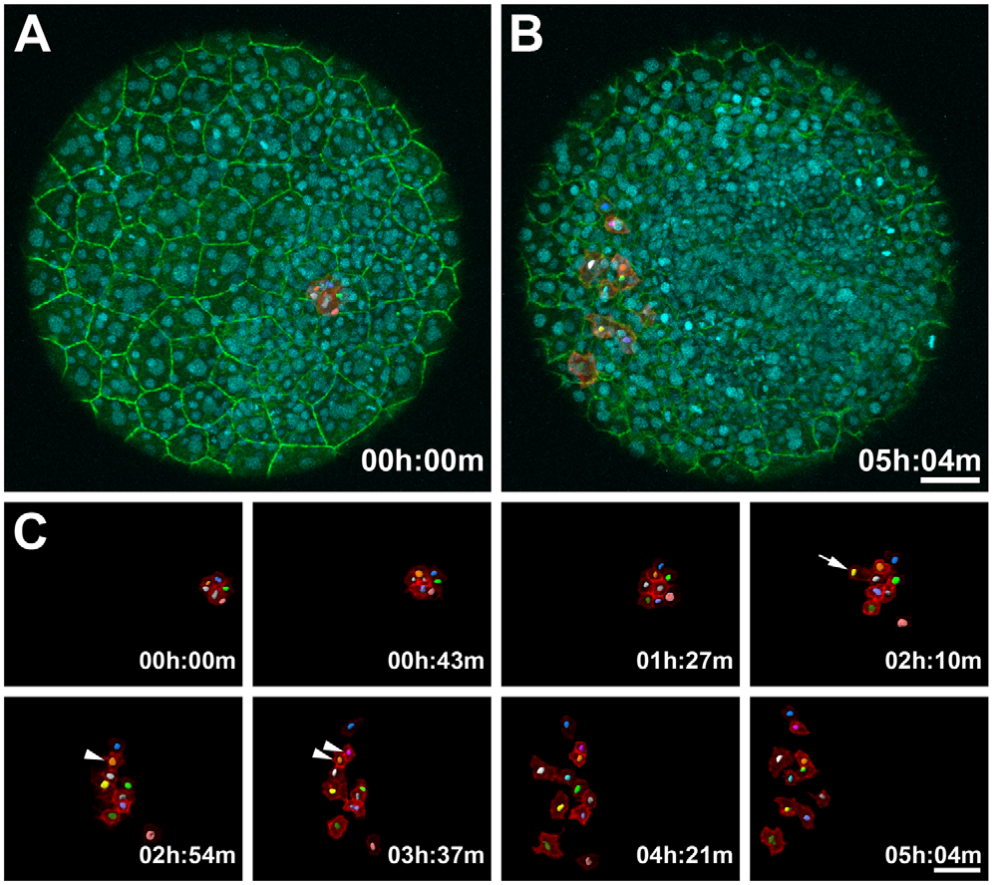
Monitoring Dynamic Membrane Movements and Tracking Nuclei in a PhOTO-M Zebrafish During Gastrulation. Animal pole view maximum intensity projection (MIP) images of the first 21µm (in depth) of an ∼5 hour time-lapse of a heterozygote F1 PhOTO-M zebrafish from late gastrulation (>80% epiboly) until early segmentation. (**A**) Merged MIP of the first frame of the time-lapse, showing unconverted (green) memb-Dendra2, segmented photoconverted (red) memb-Dendra2, and H2B-Cerulean (blue) labeled nuclei as well as segmented H2B-Cerulean nuclei (multi-colored surfaces in the photoconverted cells). (**B**) Merged MIP image of the final frame of the time-lapse. (**C**) Zoomed MIP images of segmented, photoconverted, and migrating memb-Dendra2 intensity data (red) and segmented H2B-Cerulean nuclei (multi-colored surfaces) at 8 different time-points. Note that H2B-Cerulean was segmented using the surrounding membranes as a guide in three dimensions. Cells are migrating in front of the developing head and move apart laterally. Arrowheads depict a cell division event and the arrow depicts a cell moving into the field of view from below. Note that a few overlying enveloping layer (EVL) cells were photoconverted in addition to the tracked cells, but since these remained stationary throughout the time-lapse, they were neglected when performing semi-automated membrane segmentation of the data. Scale bars are 50µm.

### PhOTO-M highlights the dynamics of cell membranes and nuclei during early development

**Figure 3:**
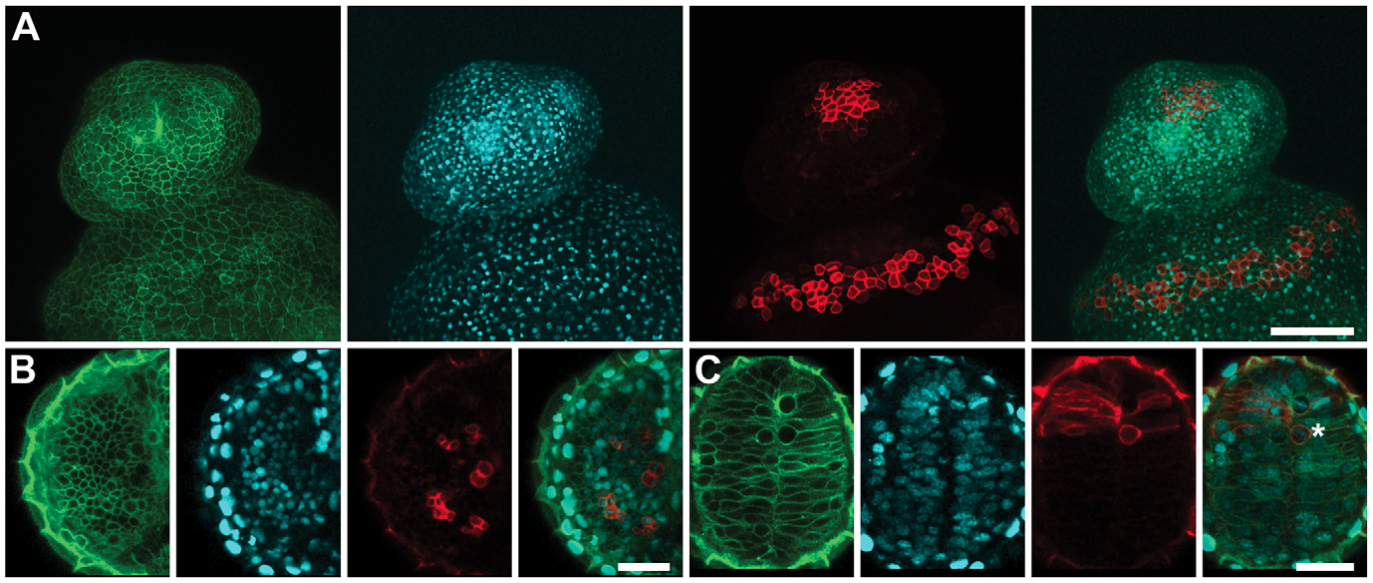
PhOTO-M Photoconversion Persists in Multiple Cell Types. Zebrafish were photoconverted in a <100µm circular region of interest near the animal pole during gastrulation and were imaged the following day to visualize cell membranes at high resolution in different cell compartments. Even after multiple rounds of division, photoconverted membranes are still clearly visible above background. (**A**) Photoconverted membranes are clearly visible in the epithelium a day after photoconversion. Maximum intensity projections of a ventrally mounted zebrafish (anterior top) embryo 1 day post fertilization. Left to Right: Unconverted memb-Dendra2 (green), H2B-Cerulean (blue), photoconverted memb-Dendra2 (red) and a merged image of the three channels. (**B**) Photoconverted membranes are visible and are separated by unconverted membranes in the eye a day after photoconversion. Single focal plane images of the developing eye in a ventrally mounted zebrafish (anterior top) embryo 1 day post fertilization. Left to Right: Unconverted memb-Dendra2 (green), H2B-Cerulean (blue), photoconverted memb-Dendra2 (red) and a merged image of the three channels. (**C**) Photoconverted membranes are visible in the developing forebrain a day after photoconversion. Single focal plane images of the developing forebrain in a ventrally mounted zebrafish (anterior top) embryo 1 day post fertilization. The star indicates an individual photoconverted cell in mitosis during prometaphase. Left to Right: Unconverted memb-Dendra2 (green), H2B-Cerulean (blue), photoconverted memb-Dendra2 (red) and a merged image of the three channels. Scale Bar in (A) is ∼150µm. Scale Bar in (B) and (C) is ∼30µm.

To extend lineage tracing beyond the level of nuclei alone, we utilized the PhOTO-M line to capture a condensed, high-resolution view of cell dynamics at the membrane level (**Figure 2** and **Videos S3, S4**). Although gross membrane morphological changes (e.g. total cell volume changes) in all cells are visible in the unconverted memb-Dendra2 channel, small-scale membrane dynamics of cells within the embryo can be appreciated only in a sparsely labeled environment such as in the segmented (see methods) photoconverted memb-Dendra2 channel, where short membrane extensions can be seen to reach out as the cells crawl across the surface of the embryo. We also used the photoconverted membranes as visible boundaries to segment the H2B-Cerulean labeled nuclei within these cells, allowing us to distinguish cell division events (arrowheads in **Figure 2C**) and cell migration into the field of view (arrow in **Figure 2C**) with high precision. Photoconverted membranes are still visible above background greater than 20 hours after photoconversion in different cell types (**Figure 3**), ensuring the documentation of membrane behavior throughout early embryogenesis as well as during major tissue specification events later in development.

### PhOTO-N enables identification of cell contributions to zebrafish epimorphic tail fin regeneration

**Figure 4:**
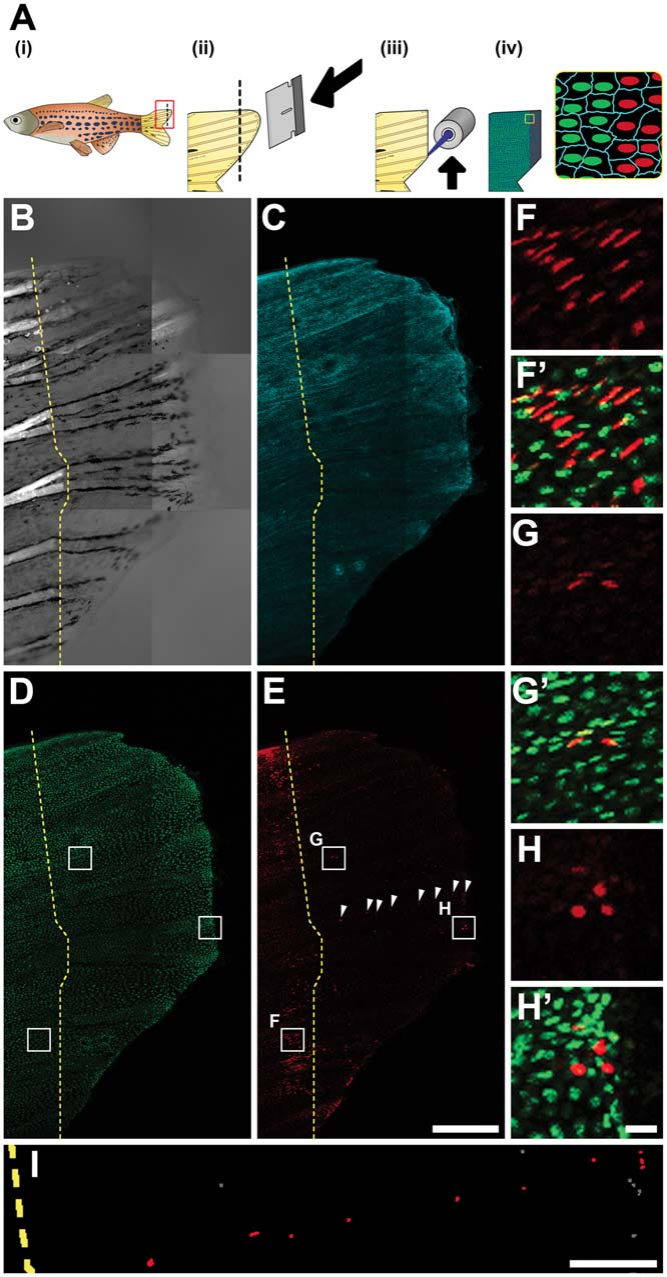
Photoconverted Cell Organization After Epimorphic Tail Fin Regeneration in a Living Adult PhOTO-N Zebrafish. (**A**) Overview of fin regeneration protocol. (**i**) An adult zebrafish is anesthetized and (**ii**) the tail is amputated using a razor blade (dotted line refers to amputation plane). (**iii**) Photoconversion is achieved by fluorescent illumination along the amputation plane. (**iv**) In addition to the H2B-Dendra2 (green) and memb-Cerulean (blue) signal, there is now a line of photoconverted H2B-Dendra2 nuclei (red). (**B-I**) Lateral view (anterior left, ventral down) MIP images of the regenerating tail fin of a live, anesthetized adult zebrafish 7 days post amputation. The dotted yellow line indicates the approximate amputation plane. (**B**) Bright field channel. Bright signal to the left of the dotted line arises from tissue birefringence. The terminal end of this signal indicates the amputation plane. (**C**) Memb-Cerulean (blue) and (**D**) unconverted H2B-Dendra2 (green) channels indicate that expression is still consistent as the tail fin regenerates. (**E**) Many of cells with fluorescence above background appear both before and after the plane of amputation in the photoconverted H2B-Dendra2 (red) channel. Cells in the central region of the tail show the least expression, suggesting extensive cell division contributing to regeneration in this area. However, certain cells to the right of the amputation plane are bright, aligned, and evenly spaced (arrowheads). (**F-H**) Zoomed boxed regions from (D) and (E) of photoconverted H2B-Dendra2 (red) alone. (**F′-H′**) Merged unconverted H2B-Dendra2 (green) and photoconverted H2B-Dendra2 (red) within the zoomed boxed regions from (D), (E). (**F, F′**) Many photoconverted cells in the ventral and dorsal portions of the amputated fin stayed behind the amputation plane during the regeneration process. (**G, G′**) Surprisingly, a subset of brightly photoconverted cells were found in the central portion in addition to (**H, H′**) other bright cells at the distal edge of the regenerate. (**I**) Zoomed binary image of a subset of the segmented nuclei from the photoconverted H2B-Dendra2 channel in panel (E). Among the scattered cells with photoconverted signal over background (gray), certain cells (red) seem aligned and evenly spaced along the anterior-posterior axis of the tail fin within the regenerating region. Scale bar for (B-E) is 300µm. Scale bar for (F-H) is ∼10µm. Scale bar for (I) is ∼100µm.

Since distinct, constitutive FP expression continues throughout the lifetime of PhOTO zebrafish, these lines can be extended to approaching problems such as live imaging of epimorphic tissue regeneration. Building upon previous studies of heart [10], [11] and tail fin [12] regeneration, we used the PhOTO-N line to probe cell contributions to the regenerating tail fin (**Figure 4A**). We amputated the caudal portion of an adult (∼7 months old) PhOTO-N zebrafish tail fin (**Figure S3**), photoconverted all nuclei within an ∼100µm stripe along the amputation plane (see methods) (**Figure S4**), and allowed the fin to regenerate for 7 days. Ubiquitous memb-Cerulean and unconverted H2B-Dendra2 expression was re-established in the regenerating tissue (**Figure 4C, D**), indicating little, if any, down-regulation of the *b-actin2* promoter driving FP expression. Using the photoconverted nuclei (**Figure 4E**), we obtained localization and intensity data that act as a readout for the extent of cell migration and division, respectively. We observed a large subset of cells with bright photoconverted nuclei that sit just rostral to the amputation plane (**Figure 4F**), presumably maintaining structural integrity of the fin during regeneration. Signal from these bright photoconverted nuclei does not colocalize with unconverted H2B-Dendra2 (**Figure 4F** **′**), indicating that these cells did not divide, since the H2B protein has a long half-life [**22**] in non-dividing cells.

**Figure 5:**
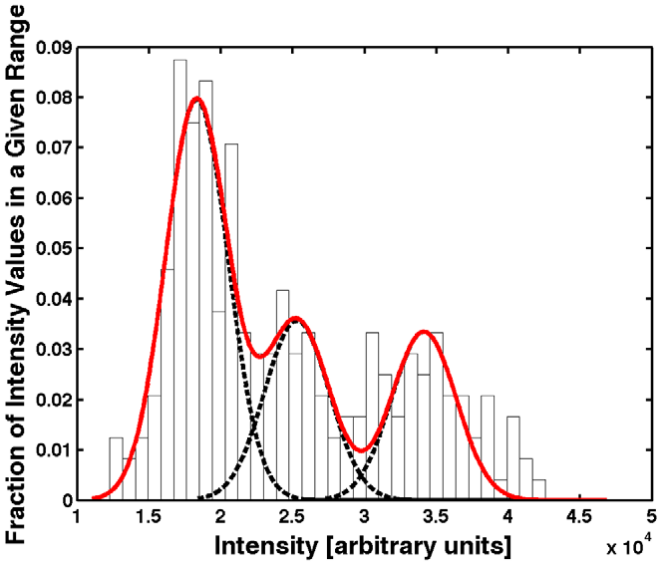
Histogram Readout for the Extent of Cell Division After 7 Days of Regeneration. Intensity data from individual nuclei within the regenerated region (posterior to the amputation plane) of the tail fin in the photoconverted H2B-Dendra2 channel (**Figure 4E**) was segmented. The average intensity of each segmented nucleus was recorded and this data was plotted as a histogram. The data was fit to a sum of 3 Gaussian curves, suggesting at least three distinct levels of fluorescence in the photoconverted cells above background. These three levels are indicative of the extent of divisions undergone during the re-establishment of the tail fin: the population with the highest average intensity underwent the fewest divisions, while the other two populations divided more often during the 7 day regeneration period.

Within the regenerated portion of the tail fin, we segmented all of the photoconverted H2B-Dendra2 nuclei that remained above background using an automated script (see methods) and discovered three distinct intensity populations (**Figure 5**). Cells with no red signal over background – presumably from the contribution of unconverted cells more rostral to the amputation plane [12] – and those with slight increases over background made up the majority of cells throughout the newly regenerating region, indicating that most of the cells in the new territory result from extensive proliferation as the fin structure is re-established. As expected, a subset of bright cells were located at the distal edge of the tail fin, consistent with previous studies [23] (**Figure 4H**). However, we also see bright cells in the middle of the regenerate (**Figure 4G**) that continue to stay visible for over 14 days (data not shown). The lack of colocalized unconverted H2B-Dendra2 (green) signal with the brightest photoconverted (red) nuclei (**Figure 4G** **′, H′**) suggests that these particular photoconverted cells managed to populate the regenerate without dividing at all. Notably, a subset of these cells was aligned and evenly spaced along the growth axis of the regenerating tissue (**Figure 4I** and arrowheads in **Figure 4E**).

## Discussion

The PhOTO lines enable non-invasive, non-random mosaic labeling of the membranes or nuclei of any subset of cells at any time during development by use of the photoconvertible property of Dendra2, combining the strengths of global and sparse/partial lineage tracing techniques and enabling selective lineage tracing during adult regeneration. The photoconversion is immediate and nontoxic, circumventing experimental shortcomings seen with heat shock or drug inducible promoters for mosaic labeling, which require significant incubation time before expression and have possible cytotoxic effects during temperature or drug treatment. An additional feature is that the multi-compartment labeling in the PhOTO lines eliminates the need for exogenous counterstains.

### PhOTO zebrafish and recent lineage tracing methods

Current analyses of early tissue specification are often facilitated by the use of tissue specific promoters driving FP expression [24]. In many situations, tissue specific transgenic lines expressing FP reporters exist and represent attractive tools to quickly identify a region of interest to focus on for lineage analysis. To target subpopulations of tissue specific promoter driven sequences, clever methodologies have been devised, including combining tissue specific FP expression with clonal mosaic analysis [5] or with mutant analysis [25]. As a complementary approach, when tissue specificity and instantaneous targeting of subpopulations of cells within labeled tissues for lineage analysis is desired, either PhOTO transgenic line may be incrossed with the tissue specific line of interest. The sole constraint for applying this PhOTO incross method for lineage analysis is that the expressed FP from the tissue specific transgenic should not have significant spectral overlap with the photoconverted Dendra2 channel.

Additionally, a useful non-transgenic labeling approach for zebrafish lineage tracing has been described recently, the so-called SNAP-Tag [26]. Like the PhOTO lines, SNAP-Tags can be targeted to different cell compartments (e.g. nuclei and membranes) and may be designed for photouncaging experiments. It is unclear, however, how long injected SNAP-Tag fluorescent conjugates will remain visible within the zebrafish, which may preclude lineage tracing past early developmental time windows. Thus, SNAP-Tags and PhOTO zebrafish are both valuable tools for early developmental lineage tracing, while the PhOTO zebrafish also has the advantage of lineage tracing during any stage in the lifetime of the zebrafish.

### Implications of PhOTO zebrafish for large-scale, systems-level analyses of cell behavior

Considerable efforts have been made to segment and track dense cell populations during development using nuclear labeling [14], [15], [27], though density and spacing of segmentable features as well as time resolution between frames increase uncertainty of these cell tracking experiments [28]. Regional reduction in segmentation and tracking complexity, which is possible with the PhOTO lines, can decrease computational effort required for large-scale embryo reconstruction, especially when selecting out areas of high cell density and mobility, as in the shield of early developing embryos. Since both the membrane and nuclear compartments are labeled, segmentation errors that occur when nuclei are in close proximity with each other can be avoided [29]. Finally, data taken within separate photoconverted regions from different embryos – especially in the case of the PhOTO-M line where small scale membrane dynamics are visible in photoconverted subsets – can ultimately be superimposed to get a sum-of-parts view of cellular dynamics during particular stages of development or regeneration, effectively eliminating uncertainty from any given region in a whole-embryo context.

Additionally, technological advances in imaging may be applied to improve resolution in both time and space for cell tracking (e.g. a recent lateral line lineage tracing study [30]). As mentioned, the PhOTO zebrafish have the advantage of targeted regional specificity when a specific transgenic line is unavailable, and they may also be used in conjunction with sophisticated imaging modalities – such as with modified selective plane illumination microscopy techniques [31], [32] – to enhance cell tracking.

### Revealing the extent of cell division using PhOTO-N zebrafish

Segmentable signal from a subset of photoconverted nuclei in the PhOTO-N line persists for at least 14 days, which makes the line an optimal tool for tracking of cells during organ formation as well as major regenerative processes in multiple tissue types (e.g. tail fin [12], heart [10], [11] and maxillary barbel [33]). Thus, any cell and its progeny can be traced from its origin before amputation to newly established regions within the regenerate, giving insight into the contributions of individual cells when repopulating a damaged area. For instance, having non-dividing cells within the regenerating region of the tail fin may suggest that cells important for guidance during the tissue regeneration process are maintained at some frequency throughout the re-established region and not solely at the leading edge. This observation seems even more plausible when considering the alignment of cells in the regenerating tissue. Such an organization seems reminiscent of the evenly spaced arrangement of cells during posterior lateral line development, where the establishment and maintenance of certain signaling cues – some of which are integral to fin regeneration [34] – guide cell migration and deposition [35]. It will be interesting to explore whether similar spatially restricted signaling contributes to the establishment of the regenerating fin.

### PhOTO zebrafish – further applications

The complexity of cell behaviors during other cell process may be ultimately unraveled due to the high signal-to-noise ratio of photoconverted fluorescence in the context of global labeling when using the PhOTO lines. Recall that three distinct cell populations could be distinguished in the regenerate, and, surprisingly, the PhOTO-N zebrafish proved to be especially suited to identify cells that did not divide during the regeneration period. As a potential extension, slowly dividing stem cells and possibly cancer stem cells may be selected from the general population of cells in a similar manner in the adult PhOTO zebrafish; photoconversion of a region of interest within a tissue followed by a several day recovery period should allow the identification and subsequent time-lapse imaging of cells within the photoconverted tissue that have divided the least.

With the combination of targeted cell tracking and global cell monitoring, the effects of therapeutics on cell behavior may also be monitored in real time. For example, since cancer stem cells have been suggested to resist traditional chemotherapies [36], photoconversion of a zebrafish tumor followed by chemotherapeutic treatment may enable time-lapse visualization of tumor regrowth from a subset of tumor cells unaffected by the treatment. Similarly, tracking cells of interest after any drug treatment that results in cell behavior changes at the single cell level (e.g. loss-of-function studies using retinoic acid [37] or morpholino oligonucleotides [38] for manipulating zebrafish regeneration) is also possible using the PhOTO zebrafish lines.

## Materials and Methods

### PhOTO Constructs

The DNA constructs were generated based on a zebrafish expression vector containing a minimal *β-actin2* promoter [16], flanking *Tol2* transposable elements [21], and a polyadenylation sequence. The TaV *2A* sequence was generated using polymerase chain reaction primer extension and further subcloned with PacI and SnaBI restriction sites. A glycine-serine-glycine spacer, which has been demonstrated to ensure high cleavage efficiency of the *2A* [39], was inserted immediately upstream of the *2A* sequence. Restriction sites were subcloned into the original zebrafish expression vector to facilitate easy modification of any element. The bicistronic template vector was generated using this vector by first incorporating a TaV *2A* sequence between the promoter and polyA sequences by subcloning. The FP Dendra2 [17] or Cerulean [18] was localized to the membrane using two repeats of a myristoylated and palmitoylated N-terminal MGCIKSKRKDNLNDDE signal sequence from Lyn kinase [19]. For nuclear localization, the C-terminus of the H2B protein was fused with either Dendra2 or Cerulean as previously described [40]. The fused Dendra2 and Cerulean constructs were subcloned with restriction sites for insertion into the PhOTO vector (sites shown in **Figure 1A**).

### Zebrafish Husbandry

Zebrafish were raised, hatched, injected, and maintained in a house colony as previously described [41].

### Zebrafish Transgenics

To establish transgenic zebrafish lines (*Tg(βactin2:memb-Cerulean-2A-H2B-Dendra2)^pw1^* and *Tg(βactin2:memb-Dendra2-2A-H2B-Cerulean)^pw2^*), WT zebrafish were first injected with either 20ng PhOTO-N or PhOTO-M plasmid DNA and 80ng *Tol2* transposase mRNA at the zygote stage. Translated Tol2 transposase proteins recognize *Tol2* elements flanking the coding region of the construct during the early developmental phases of the zebrafish, and the coding region may be inserted randomly into a cell's genome by one of these transposase proteins [21]. Injected fish were raised at 28°C until 7 days post fertilization, when they were screened for fluorescence. Healthy-looking, brightly expressing mosaic embryos/larvae from the injected population were selected and raised to adulthood. Fish that survived to adulthood were crossed to WT adults, and the resulting embryos were screened for fluorescence. Founders positive for germline transmission and for strong, ubiquitous expression were crossed with WT zebrafish to continue to propagate the line. Embryos from these founder crosses were also used for imaging.

### Western Blotting

Screened larval zebrafish from each PhOTO line as well as WT control fish were collected and homogenized in ice-cold lysis buffer (150mM NaCl, 10mM Tris-HCl at ∼pH 7.55, 1mM EDTA, 1% Triton X-100) with a protease inhibitor (Complete Protease Inhibitor Cocktail Tablets, Roche). Extracts from ∼2 fish per lane underwent SDS-PAGE (5% stacking gel/12% running gel) electrophoresis using a Mini-Protean Gel Doc (BioRad). The resulting bands were transferred to a PVDF membrane (Immobilon P, Millipore) after wet electroblotting (transfer buffer: 25 mMTris, 192mM glycine, 20% Methanol, 0.1% SDS, pH 8.1–8.5) using the Mini-Protean Gel Doc (BioRad). Rabbit anti-Dendra2 polyclonal antibodies (1:5000, Evrogen) and mouse anti-α-Tubulin monoclonal antibodies (1∶10000, Sigma Aldrich, loading control) were used to probe the western blots. Horseradish peroxidase linked goat anti-rabbit was used as a secondary antibody (1∶10000, Jackson ImmunoResearch Laboratories, Inc.). Western blots were visualized using an ECL Plus chemiluminescence kit (GE Healthcare).

### Imaging and Dendra2 Photoconversion

After crossing founder PhOTO zebrafish with WT adults, embryos were raised in egg water [41] at 28°C until they were ready to be imaged. Prior to imaging, embryos were screened for fluorescence using an Olympus MVX10 fluorescence microscope. Positive embryos were embedded in 1% low melting point agarose (Invitrogen) in 30% Danieau's solution [42] within Lab-Tek 2-well imaging chambers with #1 coverslip bottoms (Nalge Nunc International). Embryos past 16 hours post fertilization were anesthetized using 0.015%–0.03% Tricaine methanesulfonate (Finquel/MS-222, Argent Labs) and were maintained in anesthetic at the same concentration when embedded in the 1% agarose/30% Danieau's solution for imaging. We obtained live images in space and time using a 20x/0.8NA Plan-Apochromat air objective (Zeiss) using a Zeiss LSM 710 confocal microscope. Embryos were maintained at a temperature between 28°C and 32°C during time-lapse experiments. The tiled image in **Figure 1** was taken with a Leica True Confocal Scanner SP5 Spectral High-Speed Confocal System with AOBS (Acoustical Optical Beam Splitter) (Leica Microsystems Inc., Deerfield, IL) using a 20x/0.7NA objective (Leica). Photoconversion was achieved by prolonged (>30sec) illumination of a region of interest within the zebrafish sample with a scanned 405nm laser on the Leica confocal microscope. Images and time-lapse data from embryos were processed and cell segmentation and tracking was performed in a semi-automated manner using the spot tracking tool (for PhOTO-N nuclei) and the surface segmentation tool (PhOTO-N membranes and PhOTO-M membranes and nuclei) within Imaris software (Bitplane AG).

Adult zebrafish (∼7 months old) were anesthetized as mentioned above and tail fins were amputated using a razor blade. Note that only zebrafish with short caudal fins were considered for this analysis. Fast photoconversion within an hour of amputation was achieved using a 405nm excitation filter cube in the path of an X-cite mercury source (Zeiss) on a Zeiss 510 inverted confocal microscope. Photoconversion was confined using an iris in the path of the fluorescent light, and the stage was moved so that the confined conversion area was scanned along the plane of amputation [43]. Images in **Figures S3** and **S4** were taken with an Olympus MVX10 fluorescence microscope. After imaging, the fish was immediately revived using a method reported previously [44] and was then put back on the fish husbandry system (Aquaneering). For confocal imaging of the tail after 7 days, the tail was restrained under anesthesia using 2% low melting point agarose (Invitrogen) in 30% Danieau's solution. To keep the fish alive during imaging, either system water or 30% Danieau's solution containing 0.03% Tricaine methanesulfonate was flowed across the gills of the anesthetized fish, and gill motion was monitored constantly. Confocal images were taken using the same Zeiss LSM 710 confocal microscope. All images and videos were processed and compiled using Adobe Photoshop CS3 (Adobe Systems).

### PhOTO-N Nuclei Segmentation and Cell Division Analysis After 7 Days of Regeneration

Photoconverted H2B-Dendra2 MIP data from **Figure 4E** was first processed in Photoshop CS3 (Adobe Systems) to isolate the regenerated tail fin portion from the tail fin region rostral to the amputation plane using the magnetic lasso tool. Using a custom Matlab (Mathworks) script, nuclei were segmented that had intensity above background, and the segmented mean intensity data from each nucleus was fit to a sum of three Gaussians in a manner similar to one described previously [45] to generate **Figure 5**. Note that the segmented data was converted to a binary image and modified in Photoshop CS3 (i.e. changing color of the binary nuclei from white to red and gray) in order to generate **Figure 4I**.

## Supporting Information

Figure S1 **Efficient TaV2A-Mediated Protein Cleavage in the PhOTO Zebrafish Lines.** Western blot analysis of each PhOTO line: lane (1) WT (2) PhOTO-M (3) PhOTO-N. The membrane was probed for Dendra2 as well as α-Tubulin (loading control, upper blot). No Dendra2 was seen in the WT (lane 1), and each PhOTO lane had the Dendra2 protein at approximately the molecular weight expected from the particular fusion protein. Note that for lane 2, two bands are present, reflecting the post-translational palmitoylation/myristoylation additions. Efficient protein cleavage as a result of the TaV *2A* sequence was confirmed by the lack of an uncleaved product within the two PhOTO lanes. (absence of a band above 50kDa in the lower blot).
(TIF)

Figure S2 **Nuclear Photoconversion and Segmentation in a PhOTO-N Zebrafish During Gastrulation.** Animal pole view maximum intensity projection (MIP) images of the first 10.5µm (in depth from the animal pole) of an ∼6 hour time-lapse of a heterozygote F1 PhOTO-N zebrafish from late gastrulation (>80% epiboly) until early segmentation. (**A**) Merged MIP of the first frame of the time-lapse, showing memb-Cerulean (blue) and both unconverted (green) and segmented photoconverted (red) H2B-Dendra2. (**B**) Merged MIP image of the final time frame of the time-lapse. (**C**) Zoomed in MIP areas at four different time-points of the merged fluorescence images as a reference for the photoconverted images seen in panels (D) and (E). (**D**) Intensity images from segmented photoconverted nuclei (red) for the same 4 timepoints as the merged image in panel (C). (**E**) Segmented nuclei from the intensity images from panel (D). Two enveloping layer (EVL) cells are photoconverted (the orange and red spheres) as well as an epiblast cell (the white sphere). Each cell undergoes a single cell division during the course of the time-lapse. Note that the epiblast cells move beneath the field of view in the last frame, due to the development of the head during early segmentation. Scale bars are 50µm.
(TIF)

Figure S3 **Amputation of Adult PhOTO-N Zebrafish Before Photoconversion.** (**A**) A digital camera photo of the anesthetized PhOTO-N zebrafish after amputating a small portion of the upper half of the tail fin. (**B**–**E**) A fluorescent stereomicroscope image of the amputated tail fin (anterior left, ventral down) showing (**B**) bright field, (**C**) memb-Cerulean (blue), (**D**) unconverted H2B-Dendra2 (green), and (**E**) background signal when imaging with the same fluorescence emission filter as photoconverted H2B-Dendra2 (red). Note that there is almost no background fluorescent signal in (E), except for a small bit of detritus in the water that is picked up in the red fluorescence channel. Scale bar for (B–E) is ∼300µm.
(TIF)

Figure S4 **Amputated Adult PhOTO-N Zebrafish Tail Fin After Photoconversion.** A fluorescent stereomicroscope image of the photoconverted amputated tail fin (anterior left, ventral down) showing (**A**) bright field, (**B**) memb-Cerulean (blue), (**C**) unconverted H2B-Dendra2 (green), (**D**) photoconverted H2B-Dendra2 (red), and (**E**) a merged image of (C) and (D). Note that almost all of the H2B-Dendra2 fluorescence has been photoconverted in the ∼50–100µm region along the amputation plane, as is indicated by the lack of green signal in the photoconverted stripe in (C). Scale bar is ∼300µm.
(TIF)

Video S1 **Time-Lapse Data for PhOTO-N Development During Late Gastrulation.** This video shows each frame of the time-lapse summarized in **Figure S2**. (**A**) Memb-Cerulean intensity data (blue), (**B**) unconverted H2B-Dendra2 intensity data (green), (**C**) and segmented photoconverted H2B-Dendra2 intensity data (red). Initially, 3 cells were photoconverted (2 EVL cells and one epiblast). Both EVL cells divide between the first and second time point, and the epiblast cell divides between 1 hour, 19minutes and 2 hours. (**D**) Merged image of panels (A–C). Scale bar is 50µm.
(MOV)

Video S2 **Segmented Time-Lapse of PhOTO-N Development During Late Gastrulation.** This video shows segmented data for each frame of the time-lapse summarized in **Figure S2**. The nuclei were segmented: the red and orange spheres represent the EVL nuclei and the white spheres represent the migrating epiblast nuclei. The membrane of the orange-labeled EVL nucleus was segmented and is highlighted in all three panels of the image in enhanced contrast blue. (**A**) Segmented and photoconverted H2B-Dendra2 data (red) as well as the segmented EVL membrane data (blue) with the bright field data (grayscale) from the time-lapse video. The developing head and optic primordia can be seen during the time-lapse. (**B**) Segmented and photoconverted H2B-Dendra2 data as well as the enhanced contrast segmented EVL membrane data along with the merged animal pole images as seen in **Video S1D**. (**C**) Isolated zoomed images of the segmented nuclei and membranes during the time-lapse. Scale bars are 50µm.
(MOV)

Video S3 **Time-Lapse Data for PhOTO-M Membrane Dynamics Visualization During Late Gastrulation.** This video shows each frame of the time-lapse summarized in **Figure 2** in the text. (**A**) H2B-Cerulean intensity data (blue), (**B**) unconverted memb-Dendra2 intensity data (green), (**C**) segmented photoconverted memb-Dendra2 intensity data (red). Membranes are dynamic throughout the time-lapse, starting as a tight cluster of cells and eventually moving apart laterally and away from the developing head toward the ventral side of the embryo as the time-lapse progresses. (**D)** Merged image of panels (A–C), which also includes segmented nuclear data (multi-colored surfaces) within each of the photoconverted membranes. Scale bar is 50µm.
(MOV)

Video S4 **Segmented Time-Lapse Data for PhOTO-M Membrane Dynamics Visualization During Late Gastrulation.** This video shows segmented data for each frame of the time-lapse summarized in **Figure 2** in the text. First, the photoconverted membranes were segmented in 3D. Then, using the segmented data, nuclei within the surrounding photoconverted membranes were identified and represented as colored surfaces. The nuclei were tracked starting with the final frame, moving backward in time. (**A**) Segmented and photoconverted memb-Dendra2 (red) as well as the segmented H2B-Cerulean channel (multi-colored surfaces) along with the bright field (grayscale) from the time-lapse video. Note that the developing head and optic primordia can be seen during the time-lapse. (**B**) Segmented and photoconverted memb-Dendra2 (red) as well as the segmented H2B-Cerulean (multi-colored surfaces) along with the merged animal pole images as seen in **Video S3D**. (**C**) Isolated zoomed images of the segmented nuclei and membranes during the time-lapse. Scale bars are 50µm.
(MOV)
