# Supplementary figures and images for: PhOTO Zebrafish: A Transgenic Resource for In Vivo Lineage Tracing during Development and Regeneration
Source: PLoS One. 2012 Mar 14;7(3):e32888. doi: 10.1371/journal.pone.0032888 (PMC3303793; doi:10.1371/journal.pone.0032888)

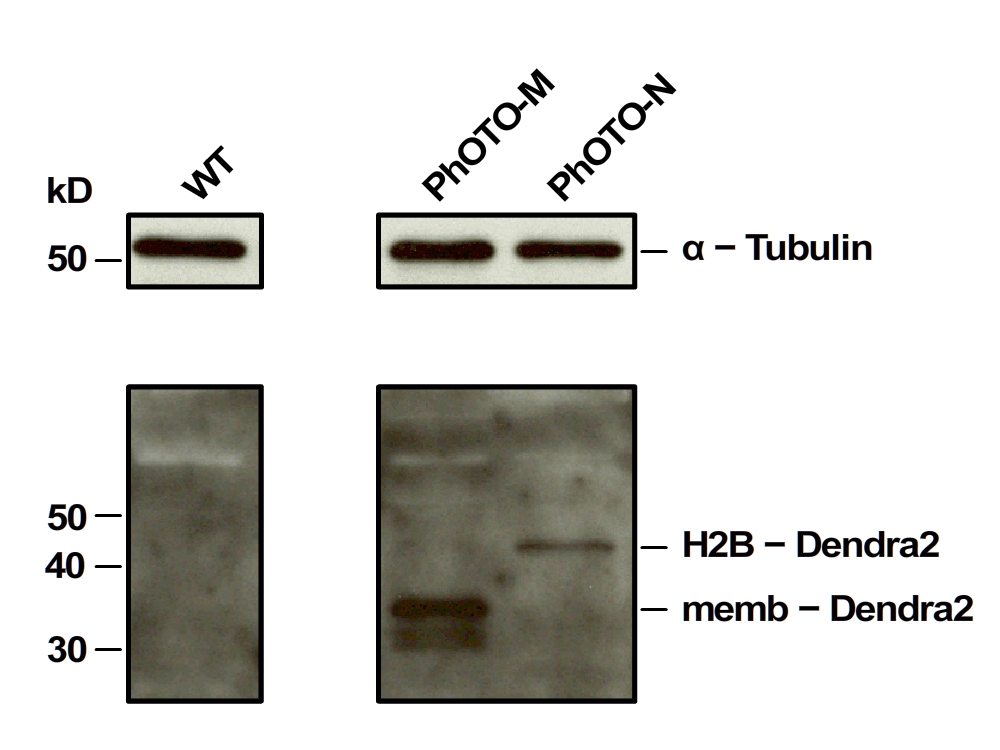

Supplement: Figure S1 — Efficient TaV2A-Mediated Protein Cleavage in the PhOTO Zebrafish Lines. Western blot analysis of each PhOTO line: lane (1) WT (2) PhOTO-M (3) PhOTO-N. The membrane was probed for Dendra2 as well as α-Tubulin (loading control, upper blot). No Dendra2 was seen in the WT (lane 1), and each PhOTO lane had the Dendra2 protein at approximately the molecular weight expected from the particular fusion protein. Note that for lane 2, two bands are present, reflecting the post-translational palmitoylation/myristoylation additions. Efficient protein cleavage as a result of the TaV 2A sequence was confirmed by the lack of an uncleaved product within the two PhOTO lanes. (absence of a band above 50kDa in the lower blot). (TIF) [file pone.0032888.s001.tif]

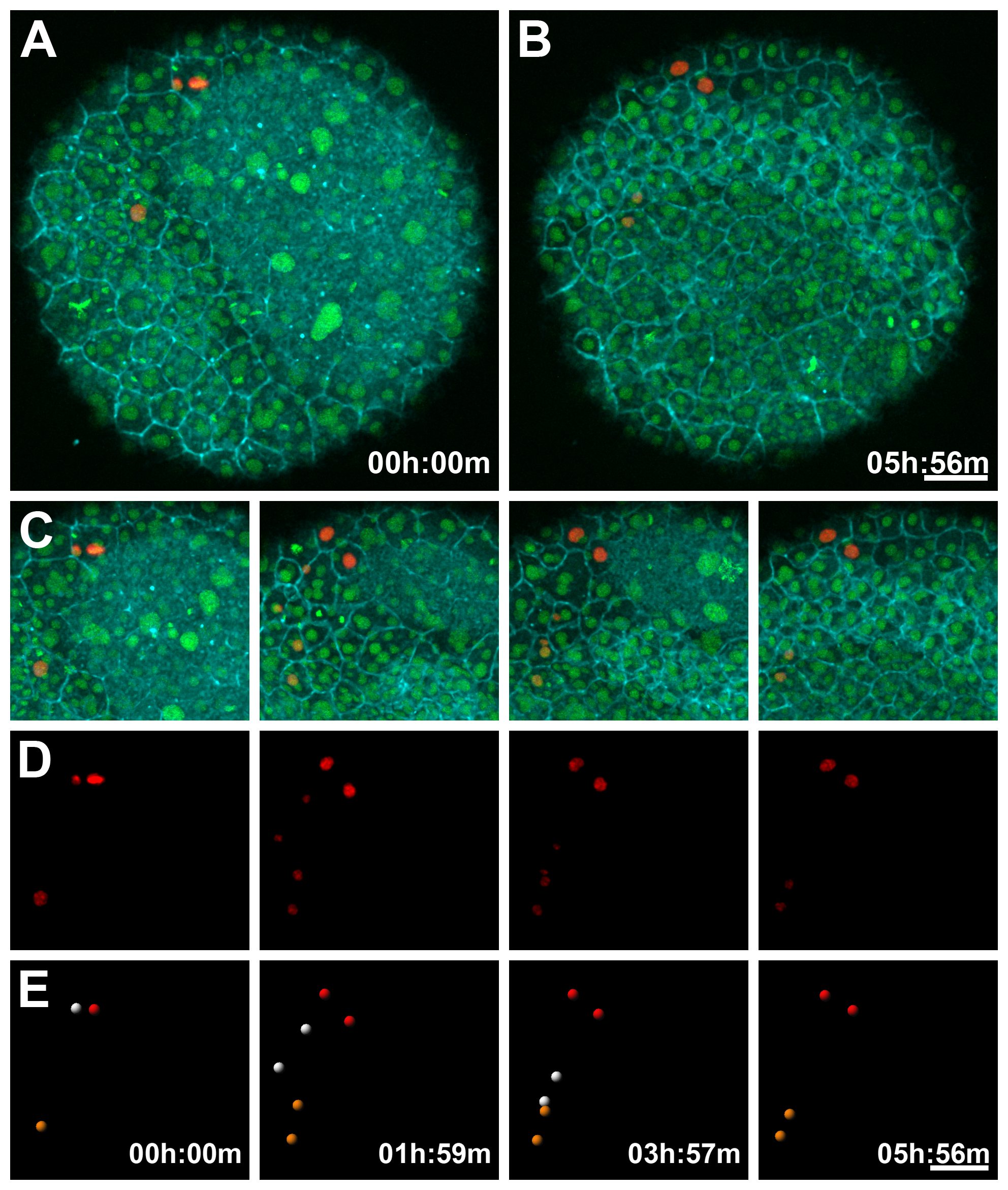

Supplement: Figure S2 — Nuclear Photoconversion and Segmentation in a PhOTO-N Zebrafish During Gastrulation. Animal pole view maximum intensity projection (MIP) images of the first 10.5µm (in depth from the animal pole) of an ∼6 hour time-lapse of a heterozygote F1 PhOTO-N zebrafish from late gastrulation (>80% epiboly) until early segmentation. (A) Merged MIP of the first frame of the time-lapse, showing memb-Cerulean (blue) and both unconverted (green) and segmented photoconverted (red) H2B-Dendra2. (B) Merged MIP image of the final time frame of the time-lapse. (C) Zoomed in MIP areas at four different time-points of the merged fluorescence images as a reference for the photoconverted images seen in panels (D) and (E). (D) Intensity images from segmented photoconverted nuclei (red) for the same 4 timepoints as the merged image in panel (C). (E) Segmented nuclei from the intensity images from panel (D). Two enveloping layer (EVL) cells are photoconverted (the orange and red spheres) as well as an epiblast cell (the white sphere). Each cell undergoes a single cell division during the course of the time-lapse. Note that the epiblast cells move beneath the field of view in the last frame, due to the development of the head during early segmentation. Scale bars are 50µm. (TIF) [file pone.0032888.s002.tif]

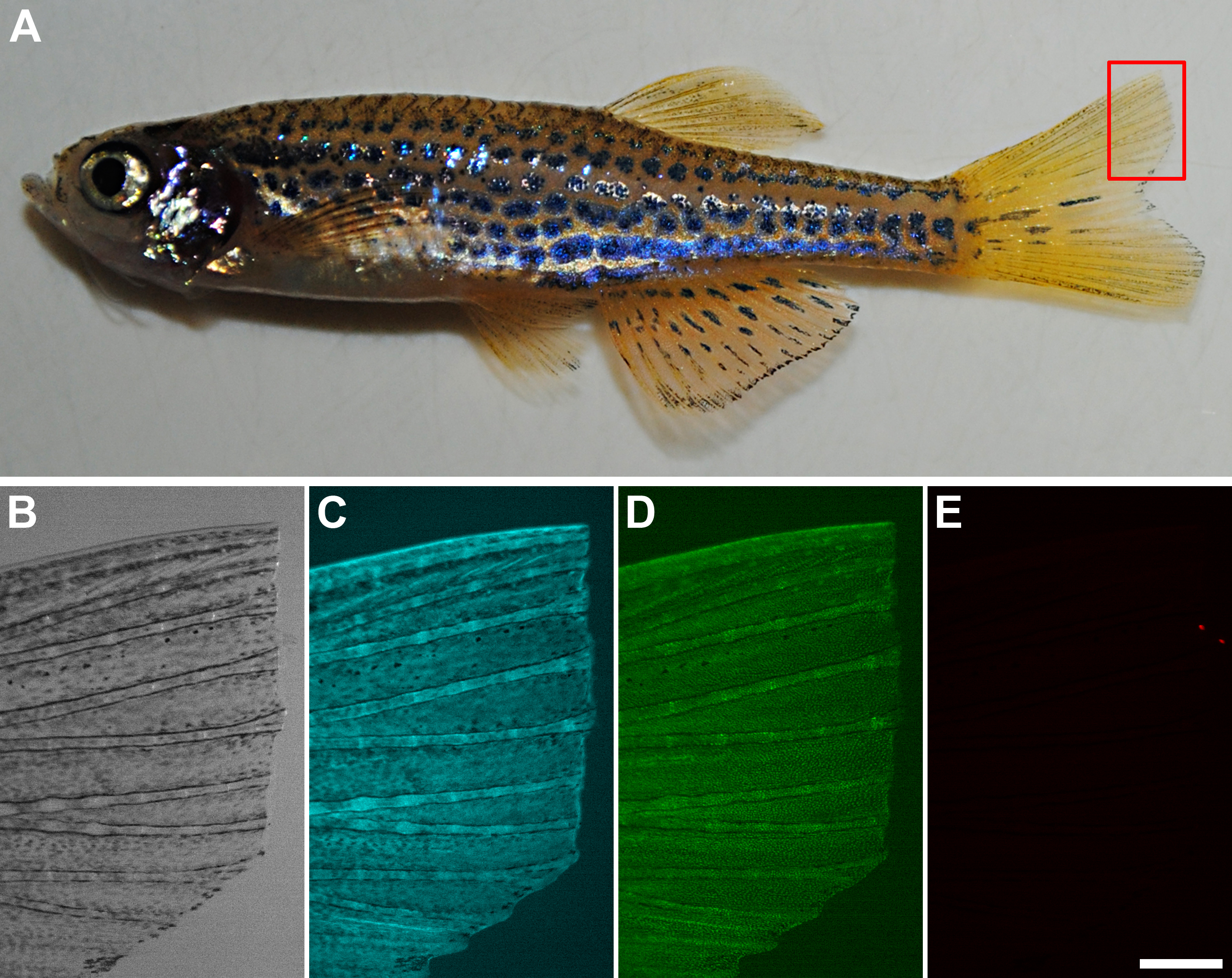

Supplement: Figure S3 — Amputation of Adult PhOTO-N Zebrafish Before Photoconversion. (A) A digital camera photo of the anesthetized PhOTO-N zebrafish after amputating a small portion of the upper half of the tail fin. (B–E) A fluorescent stereomicroscope image of the amputated tail fin (anterior left, ventral down) showing (B) bright field, (C) memb-Cerulean (blue), (D) unconverted H2B-Dendra2 (green), and (E) background signal when imaging with the same fluorescence emission filter as photoconverted H2B-Dendra2 (red). Note that there is almost no background fluorescent signal in (E), except for a small bit of detritus in the water that is picked up in the red fluorescence channel. Scale bar for (B–E) is ∼300µm. (TIF) [file pone.0032888.s003.tif]

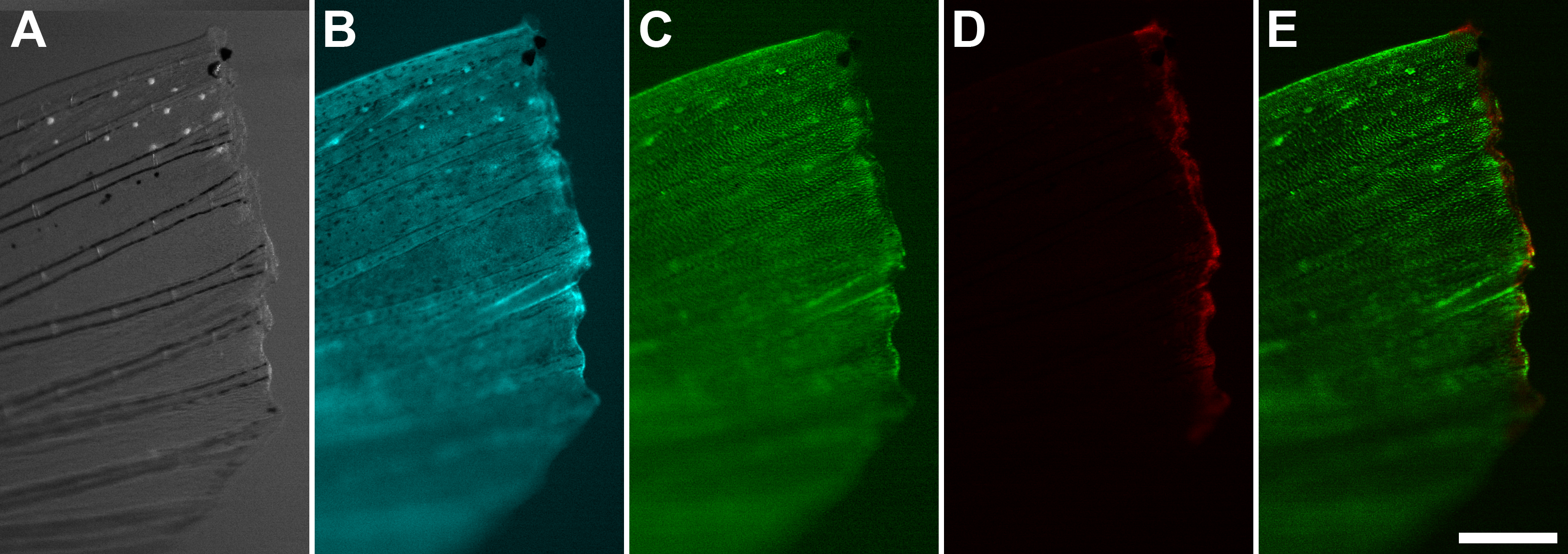

Supplement: Figure S4 — Amputated Adult PhOTO-N Zebrafish Tail Fin After Photoconversion. A fluorescent stereomicroscope image of the photoconverted amputated tail fin (anterior left, ventral down) showing (A) bright field, (B) memb-Cerulean (blue), (C) unconverted H2B-Dendra2 (green), (D) photoconverted H2B-Dendra2 (red), and (E) a merged image of (C) and (D). Note that almost all of the H2B-Dendra2 fluorescence has been photoconverted in the ∼50–100µm region along the amputation plane, as is indicated by the lack of green signal in the photoconverted stripe in (C). Scale bar is ∼300µm. (TIF) [file pone.0032888.s004.tif]
